# Development and validation of a model for staging hepatic fibrosis for chronic hepatitis B patients with E antigen-positive

**DOI:** 10.18632/oncotarget.22003

**Published:** 2017-10-24

**Authors:** Hong Wang, Ying Zhou, Rong Yan, Guo Qing Ru, Li Li Yu, Ming Shan Wang, Mei Juan Chen

**Affiliations:** ^1^ Department of Infectious Diseases, Zhejiang Provincial People's Hospital, Zhejiang, People's Hospital of Hangzhou Medical College, Zhejiang, China; ^2^ Department of Pathology, Zhejiang Provincial People's Hospital, Zhejiang, People's Hospital of Hangzhou Medical College, Zhejiang, China

**Keywords:** E antigen positive, hepatitis B virus, liver fibrosis, liver biopsy, non-invasive fibrosis marker

## Abstract

**Background:**

Interest is growing in the use of non-invasive techniques for complementing liver biopsy for liver fibrosis assessment. We aimed to prospectively evaluate liver histology in chronic hepatitis B (CHB) patients with e-antigen positivity, and develop and validate a novel scoring system—e-antigen-positive CHB liver fibrosis (EPLF) score—for noninvasively predicting the fibrosis stages.

**Methods:**

We identified the baseline variables associated with fibrosis stage (MATAVIR score, F0–F4) in 212 CHB patients with e-antigen positivity. These significant variables were used to develop the EPLF scoring system. The EPLF score equation was developed based on the prediction of fibrosis stages via multivariate ordered logistic regression analysis. The diagnostic powers of the EPLF score and several non-invasive markers were assessed through an area under the receiver operating characteristic curve (AUROC) analyses. This EPLF score model was validated in another set of 208 similar patients.

**Results:**

The natural logarithms of serum albumin, HBeAg, and HBsAg levels were selected as significant independent variables for the EPLF score equation. The EPLF score had good diagnostic power (AUROC, 0.72–0.90, p<0.001) and good diagnostic accuracy (72–85%), with a high positive predictive value (80.8–92.8%) for each fibrosis stage in the test group. Similar results were observed in the validation group (AUROC, 0.73–0.89, p<0.001). The EPLF score exhibited a strong correlation with fibrosis stage (r=0.67, p<0.001), and was the preferable non-invasive marker for staging liver fibrosis.

**Conclusion:**

In e-antigen-positive patients with CHB, the EPLF score could serve as a potential non-invasive marker of liver fibrosis stage.

## INTRODUCTION

Chronic Hepatitis B (CHB) virus infection is a public health problem. The characterization of patients with CHB is often divided into the hepatitis B e antigen (HBeAg) positive and HBeAg negative patients. It is known that HBeAg seropositive patients are at increased risk for development hepatocellular carcinoma (HCC). The severity of hepatic fibrosis is a key role for determining disease prognosis in chronic hepatitis B (CHB) patients [1, 2]. Therefore, it is important that CHB patients with significant fibrosis are diagnosed and antivirus treated early to prevent serious complications. At present, liver biopsy remains the gold standard for the evaluation of liver fibrosis stage for chronic liver disease [3, 4]. However, it is invasive and is associated with patient discomfort and with sampling errors. Therefore, the interest in the use of non-invasive techniques for complementing liver biopsy in liver fibrosis assessment is growing. Extensive resources have been invested in the past decade in the development of novel noninvasive methodologies to detect fibrosis. Many such techniques, including serum markers such as the aspartate aminotransferase (AST)/platelet radio index (APRI), Fibrotest, and the FIB-4 index, as well as transient elastograph and diffusion-weighted magnetic resonance, have been developed thus far [5, 6, 7, 8]. However, most non-invasive tests are developed from chronic hepatitis C and are accurate only when distinguishing cirrhosis from no/minimal fibrosis conditions. The use of such models in the prediction of degree of liver fibrosis in patients with chronic hepatitis B virus infection (CHB) has yielded conflicting results [8, 9, 10, 11]. In a systematic review, APRI and Fib-4 showed a moderate sensitivity and accuracy for identifying HBV-related fibrosis [12]. Moreover, other markers used in these non-invasive models may not be routinely available and be costly [13, 14]. In addition, some other non-invasion tests such as magnetic resonance require specially centers and are less available [15, 16]. Consequently development a non-invasion predictive model specified for CHB patients based on routinely available clinical parameters is priority.

Hepatitis B surface antigen (HBsAg) and hepatitis B e antigen (HBeAg) are two routine used markers to determine condition of chronic HBV infection, which were found to have inverse relationship with the severity of fibrosis in HBeAg-positive CHB patients [17, 18]. High serum levels of HBsAg and HBeAg were both found to be related with insignificant fibrosis (fibrosis stage <F2) [19, 20]. However, whether combining HBeAg and HBsAg as non-invasive biomarkers to predict fibrosis stage in HBeAg-positive CHB patients is interested.

To address the gap in knowledge, the purpose of this study was to establish a model based on serum levels of HBeAg and HBsAg, named e-antigen-positive CHB liver fibrosis (EPLF) score to predict the stage of fibrosis for HBeAg-positive CHB patients. Additionally, we evaluated its predict value in assessment fibrosis progression and comparison the predict value with that of other non-invasive fibrosis markers, including FIB-4 and APRI in validation.

## RESULTS

### Development of the EPLF score by ordered logistic regression analysis

A total of 212 patients (144 men and 68 women) met the inclusion criteria and were included in the analysis. The baseline data of the test set used in for the development of the EPLF score, stratified by histological fibrosis stage, are summarised in Table 1.

**Table 1:** Baseline data stratified by fibrosis stage for the development of e antigen chronic hepatitis B fibrosis (EPLF) score

	F0 (n=32)	F1 (n=80)	F2 (n=47)	F3 (n=20)	F4 (n=33)
Age(years)	30.9±8.8	32.2±9.2	33.73±10.43	38.6±9.97	38.36±10.36
Gender(male,%)	21(65.5)	54(67.5)	26(55.3)	15(75)	28(84.8)
Albumin(g/dL)	44.32±3.11	44.25±3.62	43.29±3.67	40.6±3.39	41.05±5.08
ALT(IU/L)	34(14-332)	54(10-639)	47(13-2302)	65(10-712)	49(23-1423)
AST(IU/L)	25(16-128)	38(16-130)	40(16-1458)	50(17-353)	46(21-1849)
TB(umol/dL)	13.1(7.7-52.4)	13.2(4.7-167)	14.9(6.1-96.1)	13.4(2.1-100)	16.9(8.7-120)
GGT(IU/L)	20.5(7-73)	21(10-295)	22(10-366)	50(17-353)	45(16-245)
AKP(IU/L)	73(22-140)	77(38-154)	81(11-207)	93(40-158)	88(32-214)
WBC(×109/L)	4.67±1.32	6.75±1.33	4.99±1.29	5.41±1.47	4.59±1.59
HB(g/L)	150±19	150±17.8	144.7±14	147.8±22.4	144.3±21.8
Platelet count	206±65	200.6±61.3	185.5±51.2	161.8±43.5	150±42.8
(×109/L)					
HBV DNA	7.8±1.0	7.7±1.0	7.3±1.4	6.7±1.3	6.4±1.2
(Log_10_copies/mL)					
HBeAg(PEIU/mL)	264.2 (1.54-384.3)	233.2 (1.79-404.5)	118.9 (1.27-291.7)	5.60 (1.29-226.1)	2.71 (1.21-216.9)
HBsAg(IU/mL)	54619 (4.31-66222.2)	35561.8 (136-72495	17048 (80.2-31622.7)	5662.2 (342-13629.1)	2469.6 (21.2-34027)
BMI	23±1.7	22±1.8	21.9±2.2	22.2±2.8	23.2±1.2

BMI, body mass index; AKP, alkaline phosphatase; GGT, γ-glutamyltransferase; ALB, albumin; ALT, alanine aminotransferase; AST, aspartate aminotransferase; HBsAg, hepatitis B surface antigen ; HBeAg, hepatitis B e antigen; WBC, white blood cell count; PLT, Platelet.

Continuous variables are presented as mean and standard deviation ; categorical variables are expressed as counts and medians.

The results of the ordered logistic regression analysis are presented in Table 2. In the univariate analyses, the natural logarithms of the HbeAg levels provided the most significant coefficients (Wald, 19.99, p<0.001). In the multivariate analysis, the natural logarithms of the serum albumin levels, HBsAg levels, and HBeAg levels at examination were selected as significant independent variables. Based on the multivariate analysis, the EPLF score equation was developed as follows:

**Table 2:** Ordered logistic regression analyses for liver fibrosis stage, F0-F4

Variable	Coefficient (95% confidence interval)	Standard error	Wald	P-value
univariate analysis				
LogeALT (IU/L)	0.05-1.72	0.43	4.32	0.04
LogeAST (IU/L)	0.02-2.05	0.53	3.69	0.06
LogeALB (g/dL)	0.70-6.75	1.54	5.8	0.02
LogeHBsAg(IU/mL)	0.13-0.53	0.1	10.49	0.01
LogeHBeAg(PEIU/mL)	0.22-0.57	0.09	20.11	<0.001
Logeage(years)	0.93-1.02	0.51	0.03	0.87
LogeGGT (IU/L)	0.02-0.85	0.22	3.4	0.07
LogeTB (umol/dL)	0.52-0.55	0.28	0.02	0.96
LogeHBVDNA(copies/mL)	1.57-1.76	0.85	0.01	0.91
LogeWBC(×10^9^)	1.06-1.14	0.57	0.03	0.96
Logeplatelet(×10^9^)	1.78-1.89	0.5	2.53	0.11
Multivariate analysis				
LogeALT (IU/L)	1.86(0.96-3.59)	0.34	3.37	0.07
LogeALB (g/dL)	2.74(2.50-0.03)	3.57	8.65	0.003
LogeHBsAg(IU/mL	0.55(0.36-0.86)	0.22	7.04	0.008
LogeHBeAg(PEIU/mL	0.44(0.28-0.67)	0.22	14.16	<0.001

GGT, γ-glutamyltransferase; ALB, albumin; ALT, alanine aminotransferase; AST, aspartate aminotransferase; HBsAg, hepatitis B surface antigen; HBeAg, hepatitis B e antigen; WBC, white blood cell count; PLT, Platelet.

EPLF score=20.28—0.42×Log_e_[HBeAg (PEIU/mL)]—4.55×Log_e_[Albumin (g/dL)]—0.34×Log_e_[HBsAg (IU/mL)].

### Diagnostic accuracy of the EPLF score

Table 3 describes the AUROC, cut-off value, positive predictive value (PPV), negative predictive value (NPV), and the diagnostic accuracy of the EPLF scores for each fibrosis stage in the test set. The EPLF score had good diagnostic power for predicting each fibrosis stage (AUROC, 0.72–0.90, p<0.001). The cut-off values were calculated as −3.54 for a fibrosis stage ≥F1, −3.17 for ≥F2, −2.14 for ≥F3, and −1.99 for F4; the score showed good accuracy in the diagnosis of each fibrosis stage (0.72–0.85). For example, a patient's fibrosis score is −2.47, his fibrosis stage may be F2 with 81% possibility.

**Table 3:** Clinical and biochemical features of the training and validation set

	Training set (n = 212)	Validation set (n = 208)	p value
Age(years)	33.9±10.08	31.7±9.12	0.04
Male(n,%)	144(67.6)	136(65.4)	0.72
Alb(g/L)	43.2±3.02	44.7±3.51	0.02
GLB	28.2±4.21	27.6±4.27	0.17
ALT (IU/mL)	49(10-2315)	52.5(10-285)	0.67
AST (IU/mL)	38(16-1849)	35(11-189)	0.17
GGT (IU/mL)	26(7-326)	30(9-227)	0.38
AKP (IU/mL)	79(11-214)	76(9-192)	0.02
TB (umol/mL)	14.1(2.4-165)	13.5(5-145)	0.70
HBV DNA (Log_10_copies/mL)	7.32±1.33	7.22±1.13	0.34
HBeAg(PEIU/mL)	150.4(1.19-404.5)	173.7(1.23-362.8)	0.83
HBsAg(IU/mL)	19774.4(4.4-71295)	23705(10.55-37865)	0.95
Genotype(B/C,n)	109/104	111/97	0.38
WBC(×109/L)	5.44±1.41	5.11±0.96	0.03
Hb(g/dL)	146.5±18.55	143.1±14.4	0.06
PLT(×109/L)	187(52-487)	171(52-354)	0.04
BMI	22.6±1.91	21.3±2.22	0.03
Fibrosis stage (n,%)			
F0	32(15)	37(17.8)	0.25
F1	80(37.6)	114(54.8)	0.001
F2	47(22.1)	31(14.9)	0.05
F3	20(9.3)	17(8.2)	0.76
F4	33(15.5)	9(4.3)	<0.001

BMI, body mass index; AKP, alkaline phosphatase; GGT, γ-glutamyltransferase; ALB, albumin; HBsAg, hepatitis B surface antigen; HBeAg, hepatitis B e antigen ALT, alanine aminotransferase; AST, aspartate aminotransferase; GLB, globulin; WBC, white blood cell count; PLT, Platelet. Continuous variables are presented as mean and standard deviation ; categorical variables are expressed as counts and medians.

### Validation of the diagnostic power of the EPLF score

From July 2014 and July 2016, 215 liver biopsies were performed. Seven patients were excluded from the study, including 3 with prior antivirus therapy, 2 with concomitant liver disease, and 2 with insufficient liver tissue for analysis. The characteristics of the validation set were similar to those of the training set, as showed in Table 4. The EPLF score model was applied to the validation set. The AUROCs for predicting each fibrosis stage (F1-F4) were 0.73, 0.83, 0.86, and 0.89, respectively (p<0.001). The score also showed good accuracy in diagnosing each fibrosis stage (0.64–0.78) in the validation set (Table 5a).

**Table 4:** Diagnostic accuracy of the hepatitis B e antigen positive liver fibrosis (EPLF) score in predicting liver fibrosis stage in training set

	AUROC	95%CI	Cutoff value	Sensitivity	Specificity	PPV	NPV	LR+	LR−	Accuracy
≥F1	0.72^*^	0.63-0.82	−3.54	72%	70%	92.8%	71.4%	1.40	0.21	0.72
≥F2	0.84^*^	0.78-0.89	−3.17	81%	72%	80.8%	82.5%	4.76	0.24	0.82
≥F3	0.90^*^	0.86-0.95	−2.14	93%	83%	90.6%	83.8%	18.03	0.36	0.85
=F4	0.87^*^	0.81-0.93	−1.99	91%	76%	90.9%	76.7%	19.58	0.60	0.79

^*^p<0.001. AUROC, area under the receiver operating characteristic; PPV, positive predictive value; NPV, negative predictive value; LR+, likelihood Ratio.

**Table 5a:** Diagnostic accuracy of the hepatitis B e antigen positive liver fibrosis (EPLF) score in validation set

	AUROC	95%CI	Cutoff value	Sensitivity	Specificity	PPV	NPV	LR+	LR−	Accuracy
≥F1	0.73^*^	0.65-0.81	−3.54	66%	82%	61.8%	78.4%	1.33	0.24	0.64
≥F2	0.83^*^	0.77-0.89	−3.17	81%	75%	80.7%	74.2%	6.05	0.50	0.76
≥F3	0.86^*^	0.80-0.91	−2.14	72%	80%	96%	67%	36.18	0.71	0.70
F4	0.89^*^	0.82-0.95	−1.99	89%	78%	88.9%	77.9%	24	0.85	0.78

^*^p<0.001. AUROC, area under the receiver operating characteristic; PPV, positive predictive value; NPV, negative predictive value; LR, likelihood Ratio.

### Predictive fibrosis progression by the EPLF score

The status of the initial liver biopsy and histological examinations three years later for 12 patients are shown in Table 5b. Case 1-2 showed the same stage of fibrosis in the initial and latest histologic examination; Case 3-12 showed worsening liver fibrosis and relatively high EPLF score. Case 10-12 showed fibrosis stage from F2 to F4.

**Table 5b:** The statue of 12 patients individually examined by pair liver histology

Case No.	Initial Histological Examination			Second Histological Examination		
	Age (years)	Fibrosis stage	EPLF score	Age (years)	Fibrosis stage	EBLF score
1	30	F0	−3.93	33	F0	−3.97
2	45	F1	−4.49	48	F1	−3.69
3	27	FO	−3.98	29	F1	−4.16
4	24	F0	−4.01	27	F1	−4.00
5	28	F0	−3.85	31	F1	−3.80
6	48	F1	−3.26	51	F2	−0.15
7	43	F2	−3.69	46	F3	0.46
8	53	FO	−3.69	56	F1	−3.84
9	49	F1	−3.51	52	F2	−3.23
10	47	F2	−3.14	50	F4	−0.13
11	54	F1	−3.49	57	F4	0.29
12	43	F1	−4.44	47	F4	−1.97

### Comparison of the EPLF score, APRI, and FIB-4

**Figure 1:**
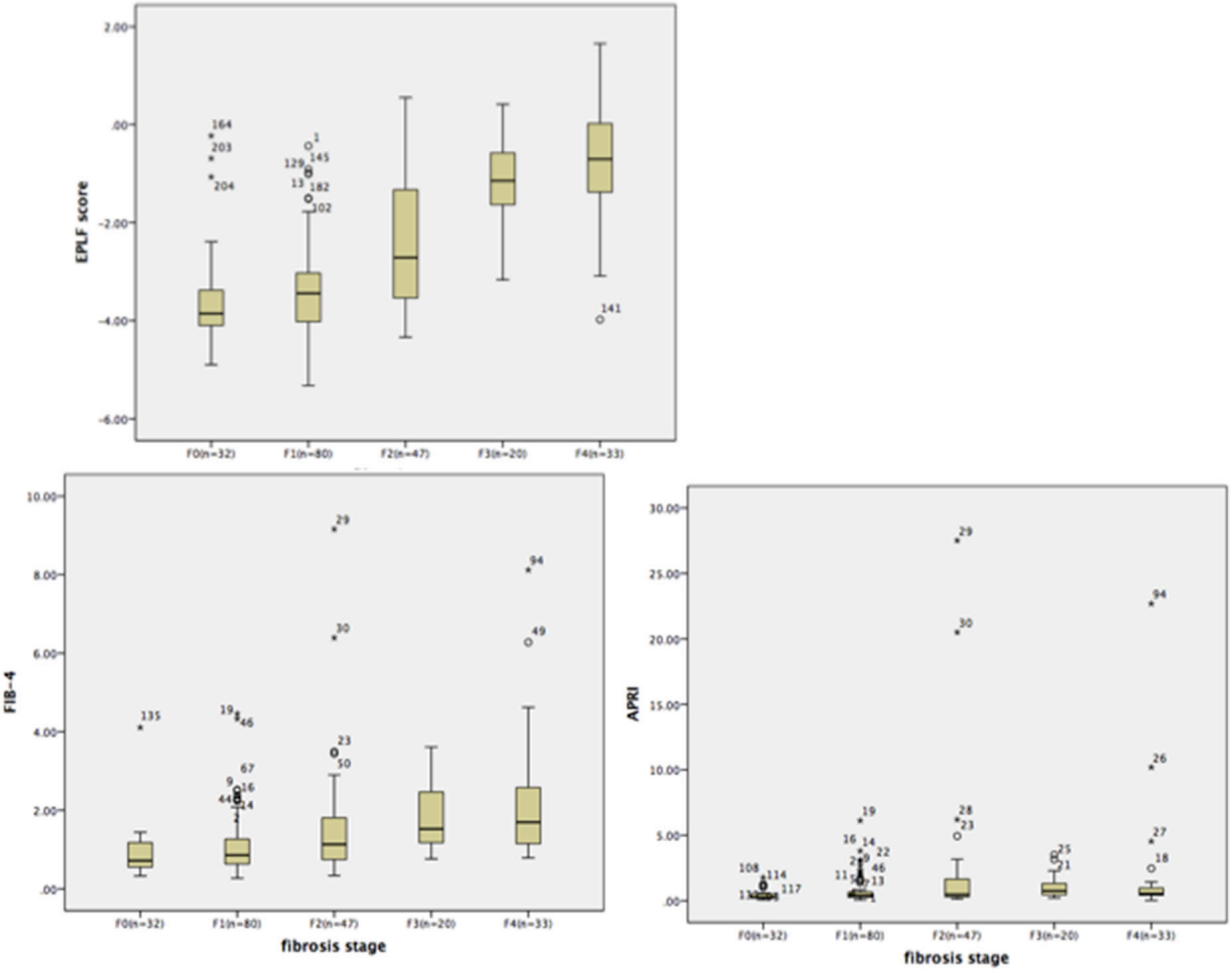
Comparisons of e antigen positive liver fibrosis (EPLF) score, FIB-4, and APRI Boxplots show the median values with the interquartile ranges, and error bars indicate the smallest and the largest values with 1.5 box-lengths of the upper and lower quartile.

**Figure 2:**
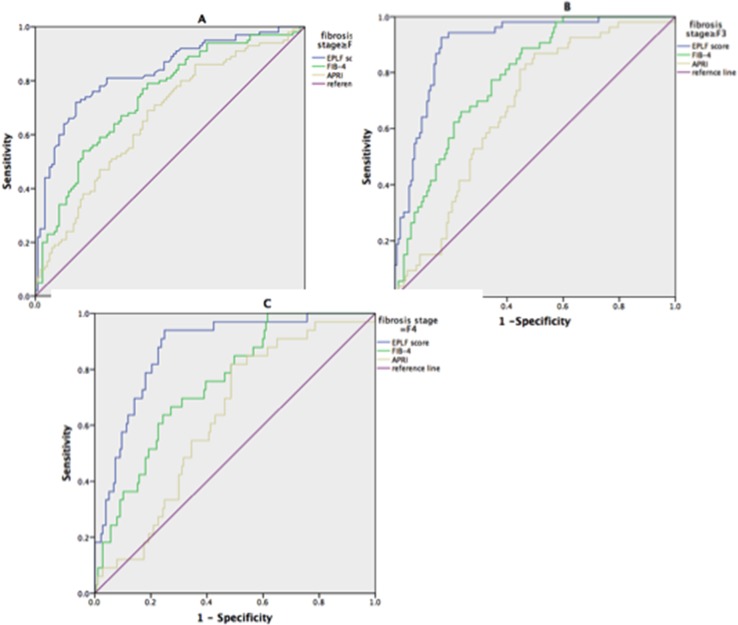
The diagnostic powers of the e antigen positive liver fibrosis (EPLF) score, FiB-4, and APRI for predicting liver fibrosis in training set **(A)** ROC plot for EPLF score, Fib-4, and APRI in differentiating significant fibrosis(METAVIR≥F2) from mild fibrosis(MATAIRE F0-F1) in the training set. EPLF score had an AUROC of 0.84, as comparing, FIB-4 and APRI had an AUROC of 0.74 and 0.66 respectively. **(B)** ROC plot for EPLF score, Fib-4, and APRI in differentiating advanced fibrosis(METARIR≥F3) from mild to moderate fibrosis(METAVIR F0-F2) in the training set. EPLF score had an AUROC of 0.90, as comparing, FIB-4 and APRI had an AUROC of 0.78 and 0.67 respectively. **(C)** ROC plot for EPLF score, Fib-4, and APRI in diagnosis of cirrhosis(METAVIR, F4) in the training set. EPLF score had an AUROC of 0.87, as comparing, FIB-4 and APRI had an AUROC of 0.75 and 0.63 respectively.

**Figure 3:**
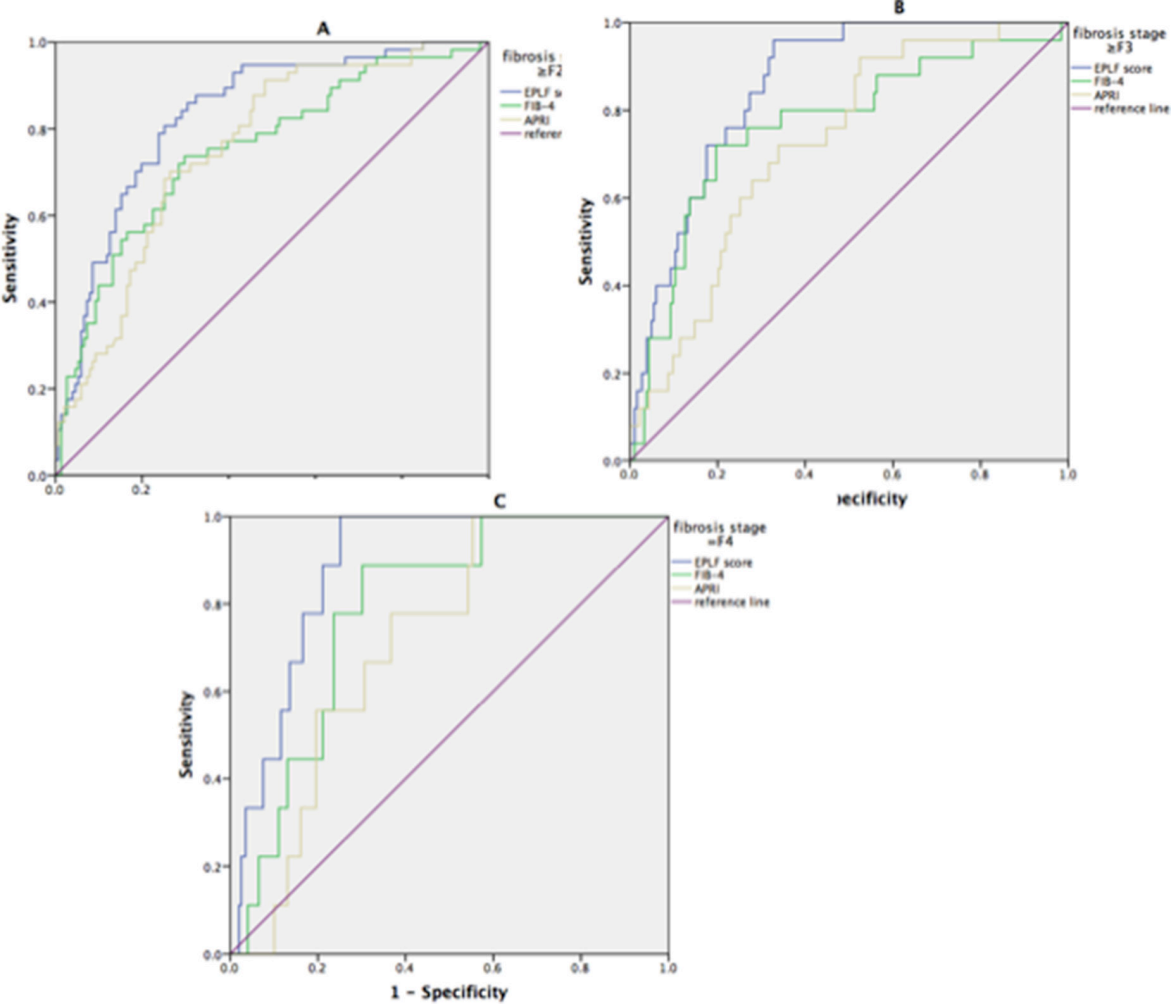
The diagnostic powers of the e antigen positive liver fibrosis (EPLF) score, FIB-4, and APRI for predicting liver fibrosis in validation set **(A)** ROC plot for EPLF score, Fib-4, and APRI in differentiating significant fibrosis(METAVIR≥F2) from mild fibrosis(MATAIRE F0-F1) in the validation set. EPLF score had an AUROC of 0.83, as comparing, FIB-4 and APRI had an AUROC of 0.75 and 0.73 respectively. **(B)** ROC plot for EPLF score, Fib-4, and APRI in differentiating advanced fibrosis(METARIR≥F3) from mild to moderate fibrosis(METAVIR F0-F2) in the validation set. EPLF score had an AUROC of 0.86, as comparing, FIB-4 and APRI had an AUROC of 0.77 and 0.73 respectively. **(C)** ROC plot for EPLF score, Fib-4, and APRI in diagnosis of cirrhosis(METAVIR, F4) in

Figure 1 shows the box plots for the EPLF score, APRI, and FIB-4 in comparison with the liver fibrosis stage. The EPLF score was most strongly correlated with the fibrosis stage (r=0.67, p<0.001), and was equally distributed from F0 to F4. The powers of the EPLF score and the other markers in diagnosing fibrosis stages ≥F2, F3, and F4—assessed via AUROC analyses in the training and validation set—are shown in Figure 2. and Figure 3. For the diagnosis of fibrosis stage ≥F2, the EPLF score had the highest diagnostic power (AUROC, 0.84, p<0.001), followed by FIB-4 (cut-off value 1.45; AUROC, 0.73, p<0.001) and APRI (cut-off value 0.50; AUROC, 0.66, p<0.001). Furthermore, for the diagnosis of fibrosis stage ≥F3, the EPLF score again had the highest diagnostic power (AUROC, 0.89, p<0.001), followed by FIB-4 (cut-off value 3.25; AUROC, 0.78, p<0.001) and APRI (cut-off value 2.0; AUROC, 0.68, p<0.001).

## DISCUSSION

In the present study, we sought to develop a model (EPLF) that employed a single point measurement of HBsAg, and HbeAg levels and liver function test results to predict the degree of liver fibrosis in a consecutive series of e-antigen-positive treatment-naïve CHB patients. We found that serum albumin., HBsAg, and HbeAg levels were independent predictors of liver fibrosis stage. A one-time baseline measurement of HBsAg, HbeAg levels and albumin levels could be used to predict liver fibrosis stage in CHB patients with HbeAg seropositive. The EPLF model including these three variables was simple to use and had comparable accuracy in staging liver fibrosis and might to be used to predict fibrosis progression in e-antigen-positive treatment-naïve CHB patients

The major finding of this study was that combined serum levels of HBeAg and HBsAg could correctly stage liver fibrosis in e-antigen-positive CHB patients with high accuracy, although the mechanisms underlying this correlation remain unclear. At some points, chronic hepatitis B infection is an immunologic tolerance disease. HBV itself has non-cytopathic towards infected hepatocytes [25]. Liver injury is mostly due to host–virus immune activity. Several important studies in recent years have shown that quantitative HBsAg levels not only correlate with covalently closed circular DNA (cccDNA) levels, they are also markers of immune control [26]. In immune active phase, the kinetics of HBsAg and HBeAg was gradually decreased. High serums levels of HBeAg and HBsAg may reflect less extended of host – virus immune activity, which may mean less extent liver injury and regeneration of hepatocytes, causing collagen deposition. Consistent with previous studies, wherein higher HBsAg levels were found to be associated with a greater likelihood of having no/mild fibrosis (F0–F1), [23, 24] Our data confirm the negative correlation between HBsAg levels and the degree of fibrosis in HBeAg-positive CHB patients. The cut-off value of 18000 IU/mL for HBsAg achieved a high AUC value (0.72) for the prediction of F0–F1. We also observed that the HBeAg cut-off value of 137.8 PEIU/mL achieved an AUC value of 0.73, and helped predicting F0–F1 (Supplementary Table 1). However, only used the levels of HBsAg or HBeAg cannot stage fibrosis.

Another finding of our study is that there is no relationship between ALT values and histologic finding in e-antigen-positive treatment-naïve CHB patients. Elevation of ALT levels is the hallmark of hepatocyte inflammation that has long been used as the best diagnostic marker for hepatitis associated with viral, drug, alcohol, or other etilogies. Because CHB patients show wide fluctuations in biochemical activities, patients with significant liver injury may experience spontaneous normalization of ALT levels for a long time. CHB patients with persistently normal ALT levels may experience severe histologic liver damage. Our results based on 420 patients found that among patients with normal ALT levels, 36%have fibrosis scores greater than F2. Our data support the reports of Kumar et al, [27] who stated that 28% to 37% CHB patients with persistently normal ALT levels may experience severe histologic liver damage. Thus, some predictive models based on ALT, AST levels such as FIB-4 and APRI may miss staging fibrosis correctly for HBeAg-positive CHB patients with normal ALT levels. The area of ROC curves of FIB-4 or APRI is low in predictive degree of fibrosis for these patients. This finding is consistent with Kim et al. report [29]. Compared with the APRI and FIB-4 score, the cohort used to develop the EPLF score system included patients with normal or mildly elevated ALT levels (ALT ≤2xULN) and patients with elevated ALT levels (ALT >2xULN). Using the EPLF score system would correctly classify 80.7% of patients with fibrosis stage ≥F2 and thus avoid biopsy. The EPLF score appears to be suitable for all patients, irrespective of the ALT levels. Furthermore, we observed that serum albumin levels of at examination showed a significant negative relationship with the fibrosis stage. The decrease in the levels of serum albumin, which is the result of reduced liver synthetic capacity, may be a marker of the high frequency of liver injury. In the multivariate ordered logistic regression analysis, the serum albumin levels exhibited a negative coefficient. Thus, the albumin levels in combination with viral factors (e.g., HBsAg and HBeAg) can increase the prediction accuracy.

Conceptually, serum aminotransferase level is much less important as a predictor of fibrosis progress in CHB patients during immune active phase as it fluctuated widely. As comparison, the levels of HBsAg and HBeAg change slowly, presumably as a result of long time of hepatic inflammation. The EPLF regression equation suggests that long-term liver fibrosis progression in HBeAg positive CHB patients is the results of immune control, represented by the levels of HBsAg and HBeAg. In a subpopulation of the current study, 10 of the 12 patients experienced fibrosis progress that can be found by the EPLF score change. The EPLF score appears to be suitable not only for staging liver fibrosis, but for evaluating fibrosis progress in treat-naïve HBeAg positive CHB patients.

Although the current study contains one of the largest series of HBeAg-positive, treat-naive CHB patients, there are several limitations to be noted. First, the current study used liver histology findings, obtained from liver biopsies, as reference parameters. Since biopsies are limited by sampling errors and exhibit variability, [28] the histological examination may yield certain false-positive results and some results may need to be omitted. Second, this study did not examine long-term trends of serum levels of HBeAg and HBsAg, future studies should investigate the ability of a onetime measurement to predict long-term trajectories of HBsAg and HBeAg. Thirdly, the quantitative measurement of HBsAg or HBeAg is not a routine clinical practice in some areas. Our model might be limited to be used in clinical practice. In addition, this study consisted only of genotype B and C patients; further studies in larger cohorts of patients with other genotypes would help to further validate the results of this study.

In conclusion, in the present study, we developed and validated a potential fibrosis marker for HBeAg-positive CHB patients, which was non-invasive, practical, and easily accessible. The use of this prediction score may help guide clinical decision making and potentially reduce the need for invasive liver biopsies in the management of HBV genotype B- or C-associated HBeAg-positive patients with CHB. The prediction score can be used to stage the liver fibrosis with high accuracy. This non-invasive prediction score approach may be of particular benefit in low-resource settings.

## PATIENTS AND METHODS

### Study design and setting

We conducted a prospective study atZhe Jiang Provincial People's Hospital between December 1, 2012, and December 1, 2015. The study protocol was reviewed and approved by the ethics committee of the hospital. Informed consent was obtained from each patient prior to participation.

### Study population

#### Training set

A total of 212 patients with CHB were enrolled in the study from the Liver Center of Zhe Jiang Provincial People's Hospital between December 1, 2012, and December 1, 2014. All the patients who fulfilled the following inclusion criteria were enrolled: age≥18 years; diagnosis of CHB based on HBsAg positivity for >6 months; HBeAg positivity; detectable HBV-DNA level (>10^4^ copies/mL); and no previous or concomitant anti-HBV therapy. We excluded patients with liver comorbidities, such as hepatitis delta superinfection, hepatitis C virus co-infection, chronic alcohol consumption (<30 g of pure alcohol per day), Wilson disease, human immunodeficiency virus co-infection, and auto-immune hepatitis. The scoring system was developed based on the data of the patients in the test group.

#### Validation set

A total of 208 patients were underwent biopsy between July 2014 and July 2016 were assigned to the validation set.

This study conformed to the ethical guidelines of the 1975 Declaration of Helsinki, and was approved by the ethical committee at Zhe Jiang Provincial People's Hospital.

#### Data collection

Blood samples were obtained from the cohort on the day before liver biopsy. Biochemical tests for the levels of fasting plasma glucose, total cholesterol, triglycerides, ALT, AST, alkaline phosphatase, γ-glutamyl-transpeptidase (GGT), bilirubin, and albumin, as well as complete blood count, were performed in the clinical laboratory of the hospital using commercially available assays. The levels of hepatitis antibodies, including HBsAg, HBsAb HBeAg, HBeAb, HBcAb, and anti-hepatitis C virus, were measured using Clinical Laboratory Improvement Act-approved systems, in accordance with AASLD practice guidelines. The serum HBV-DNA levels were assessed using a real-time polymerase chain reaction system (ABI7300; 55 Applied Biosystems, Foster City, CA, USA). The lower limit of detection was set at 200 copies/mL, and the linearity range was set from 200 to 20,000 copies/mL.

The levels of HBsAg were quantified using the Architect HBsAg assay (Abbott Laboratories; dynamic range, 0.05–250 IU/mL) after 1:100 dilutions. Samples with HBsAg levels of >250 IU/mL at 1:100 dilutions were retested at final dilutions of 1:1,000. Samples with HBsAg levels <0.05 IU/mL at 1:100 dilutions were retested in an undiluted condition. The serum level of HBeAg was determined by using a microparticle-based enzyme immunoassay with a commercially available kit (EIA, Abbott Laboratories, Chicago, IL, USA). The dynamic range of the assay is 0.15–300 PEIU/mL; the samples with concentrations beyond this range were diluted with foetal bovine serum to ensure linearity.

The values of 2 non-invasive fibrosis markers, APRI and FIB-4, were also determined. APRI values were calculated using the formula: AST (U/L)/upper normal limit×100/platelet count (10^9^/L). Moreover, the FIB-4 results were calculated using the formula: age (years)×AST (U/L)/platelet count (10^9^/L)×(ALT (U/L)^1/2^).

#### Evaluation of liver histology

All the patients underwent percutaneous liver biopsy under ultrasonographic guidance. Liver biopsy was performed using 16-G Trucut biopsy needles (Bard, Covington, GA, USA). The specimens were fixed, paraffin-embedded, and stained with haematoxylin and eosin (HE). A minimum of 1.5 cm of liver tissue with at least 9 portal tracts was required for appropriate diagnosis. All liver biopsies were reviewed by 1 pathologist (G.Q.R), who was blinded to the clinical characteristics of the study subjects. Liver fibrosis was evaluated according to the MATAVIR scoring system [21] as follows: F0, no portal fibrosis; F2, portal fibrosis with rare septa; F3, numerous septa or lobular distortion without cirrhosis; and F4, cirrhosis.

#### Development of the EPLF score using ordered logistic regression analysis

To predict the histological fibrosis stage, ordered logistic regression analyses were performed, by using the histological fibrosis stages as ordinal data (F0, F1, F2, F3, and F4) and as dependent variables; the independent variables included standard biochemical and haematological test results, gender, viral markers, and age. The independent variables with strong correlations (r>0.7) in the multivariate logistic regression analyses were excluded to avoid multicollinearity. The scoring system equation was developed by adding a minus sign to the regression equation for the logit of F0 probability in the multivariate analysis.

### Validation of the scoring system

After developing the scoring system equation, we measured the score in the test group during each histological examination. The diagnostic value of the EPLF score, APRI, and FIB-4 were calculated and evaluation in the validation set, and the discrimination performance was also evaluated using the validation dataset.

#### Analysis of pair liver histology and the EPLF score in 12 patients

Among the 212 patients involved in this study, 12 patients had a second histological examination after 36 months. The EPLF score and other non-invasive markers were calculated at time of each histological examination.

### Statistical analysis

Quantitative variables are expressed as means (standard deviation) in the case of normal distribution; otherwise, the data are presented as medians (interquartile range). Categorical data are presented as frequencies and percentages. The correlations between the ordinal and continuous data were assessed using the Spearman correlation coefficient. For logistic regression analysis, the p value of each independent variable was determined by the Wald chi-square value (Wald). The diagnostic powers of the EPLF score and other fibrosis markers were evaluated based on the area under the receiver operating characteristic curve (AUROC); the cut-off values were determined by maximising the sum of the sensitivity and specificity, according to Youden's index [22]. Moreover, p values <0.05 were considered statistically significant. Statistical analyses were performed using SPSS20.0 software (IBM SPSS, Chicago, IL, USA).

## SUPPLEMENTARY MATERIALS TABLE



## List of abbreviations

HBV: hepatitis B virus
DNA: deoxyribonucleic acid
CHB: chronic HBV-infection
HBsAg: HBV surface antigen
HBeAg: HBV e antigen
HCC: hepatocellular carcinoma
ALT: alanine aminotransferase
AST: aspartate aminotransferase
HBsAg: hepatitis B surface antigen
HBeAg: hepatitis B e antigen
WBC: white blood cell count
PLT: Platelet. AUROC
PPV: positive predictive value
NPV: negative predictive value
LR+: likelihood Ratio
CHB: chronic hepatitis B
EPLF: e-antigen-positive CHB liver fibrosis
APRI: the aspartate aminotransferase (AST)/platelet radio index
ROC curves: Receiver Operating Characteristic curves
