# Supplementary material for: Development and validation of a model for staging hepatic fibrosis for chronic hepatitis B patients with E antigen-positive
Source: Oncotarget. 2017 Oct 24;8(58):98812–22. doi: 10.18632/oncotarget.22003 (PMC5716769; doi:10.18632/oncotarget.22003)
Supplement: Supplementary file 1 [file oncotarget-08-98812-s001.pdf]

Development and validation of a model for staging hepatic fibrosis for chronic hepatitis B patients with E antigen-positive

SUPPLEMENTARY MATERIALS

Supplementary Table 1: Diagnostic accuracy of the hepatitis B e antigen positive liver fibrosis (EPLF) score in validation set

	AUROC	95%CI	Cutoff value	Sensitivity	Specificity	PPV	NPV	LR+	LR−	Accuracy
≥F1	0.73*	0.65-0.81	−3.54	66%	82%	61.8%	78.4%	1.33	0.24	0.64
≥F2	0.83*	0.77-0.89	−3.17	81%	75%	80.7%	74.2%	6.05	0.50	0.76
≥F3	0.86*	0.80-0.91	−2.14	72%	80%	96%	67%	36.18	0.71	0.70
F4	0.89*	0.82-0.95	−1.99	89%	78%	88.9%	77.9%	24	0.85	0.78

*p<0.001. AUROC, area under the receiver operating characteristic; PPV, positive predictive value; NPV, negative predictive value; LR, likelihood Ratio.
